# 

**Published:** 1897-06-19

**Authors:** 


					Hiiuuiju iHL.1 PUK.&, uiereiore be.s>i.  uune iyiH, isa7.
OADBURY'S m w, CADBURY'S
OOCOA INI coooa
'TtUinaW?'-ZaS.eSt ^ ^ NO ALKALIES USED.
'/JMWW HOSPLTAt "
No. 66j>. Vol. XXII. [SM Satttedat,
June 19th, 1897.
[SubsoriptionTerms,] pB(ICE TWOPENCE.

				

